# Supplementary material for: When Does the Prey/Predator Ratio Work for the Effective Biocontrol of Cotton Aphid on Cotton Seedlings?
Source: Insects. 2022 Apr 21;13(5):400. doi: 10.3390/insects13050400 (PMC9147916; doi:10.3390/insects13050400)

Figure S1. The change of daily temperatures in a garden in Xinjiang Institute of Ecology and Geography, Chinese Academy of Sciences, Urumqi, Xinjiang.

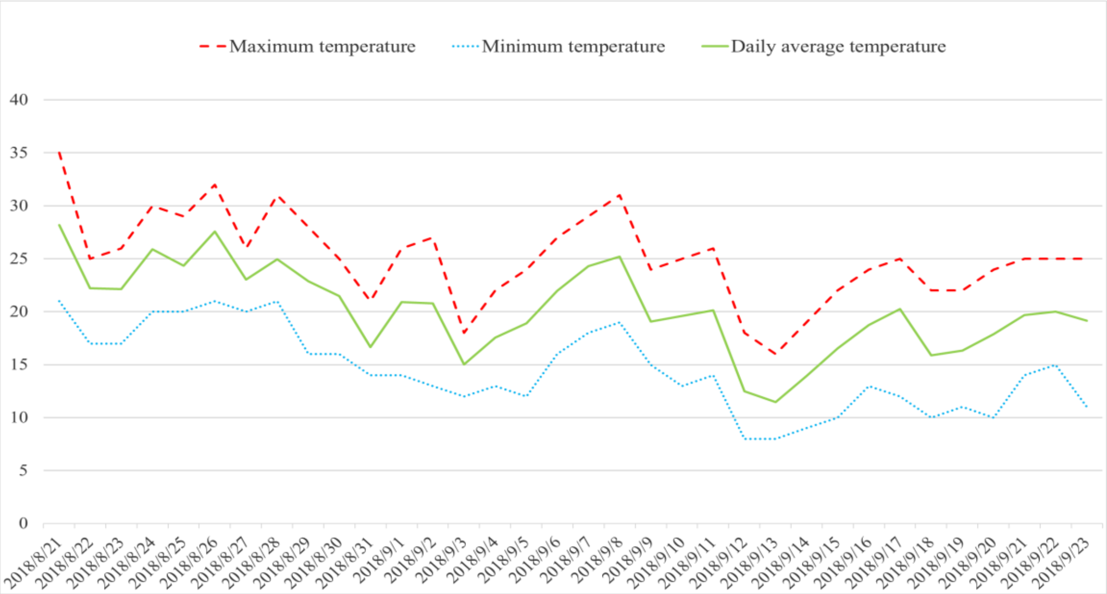

Supplement: Supplementary file 1 [file insects-13-00400-s001.zip › insects-1654672-supplementary.pdf]
